# Study on selection of native greening plants based on eye-tracking technology

**DOI:** 10.1038/s41598-022-05114-0

**Published:** 2022-01-20

**Authors:** Ningning Ding, Yongde Zhong, Jiaxiang Li, Qiong Xiao

**Affiliations:** 1 Central South University of Forestry and Technology, Changsha, 410000 China; 2 National Forestry and Grassland Administration State Forestry Administration Engineering Research Center for Forest Tourism, Changsha, 410000 China

**Keywords:** Human behaviour, Psychology and behaviour, Sustainability, Environmental impact

## Abstract

The selection of native greening plants to improve rural greening technology is crucial for enriching methods of building rural plant landscapes. However, there are few studies from the perspective of visual preference using quantitative methods. By using eye-tracking technology, this study studies students in the Central South University of Forestry and Technology and villagers in Changkou Village, Fujian Province, employing pictures of plant organs—leaves, flowers, and fruits—as stimulating materials to analyze five indicators: the total duration of fixations, the number of fixations, average duration of fixations, average pupil size and average amplitude of saccades. A number of findings came from this research First, people visually prefer leaves, followed by flowers and fruits. In terms of species, *Photinia* × *fraseri*, *Metasequoia glyptostroboides*, *Photinia serratifolia*, *Cunninghamia lanceolata* and *Koelreuteria bipinnata* have higher overall preference. Families such as *Malvaceae, Fabaceae, Araliaceae, Myricaceae* and *Cupressaceae* have stronger visual attraction than others. Second, there are distinct differences in the preference of shapes and textures of leaves: aciculiform, strip, cordiform, sector and jacket-shape are more attractive; leather-like leaves have a higher visual preference than paper-like leaves; different colors and whether leaves are cracked or not have little effect on leaf observation. Third, the preference for flowers with different inflorescence and colors is significant. Capitulum, cymes and panicles are more attractive; purple is the most preferred color, followed by white, yellow and red. Finally, there are significant differences in preferences for fruit characteristics, with medium-sized fruits and black fruits preferred, while kidney-shaped and spoon-shaped fruits are considered more attractive. Pomes, pods, samaras, and berries have received relatively more attention.

## Introduction

With rapid economic growth and urbanization, unprecedented change has come to China’s rural regions. As a result, the traditional rural landscape has been greatly disturbed, affecting agricultural production and the quality of life of rural farmers. Currently, traditional urban greening technology is used primarily in China’s rural greening programs, while the application of native plants has not attracted sufficient attention, accounting for a relatively small proportion of tree species used. There are, however, major challenges in native implementation, such as vegetation type^1^, lack of characteristics^2^ and lack of seedling sources for native plants^3^. The consequent phenomenon of “a thousand identical villages” divorces the human environment from the natural landscape of rural areas in China. In the face of emergent scenarios and new problems, research, and development of greening technology suitable for Chinese villages has become a necessary technological breakthrough for the goal of constructing “green and livable rural villages and towns”^4^.

Plants are one of the most important elements of open spaces, and of environmental perception and preference^1^. Some scholars use the term “visual environment” to describe people’s visual perception of their surroundings^2–4^, but more prefer the term “visual landscape” to highlight the visual attributes of the landscape^5–7^. The landscape described as ‘the environment’ in this context can be anything from micro-scale to macro-scale, referring to urban parks, plants^8^ and plant organs. Visual preference for landscape refers to the influence of landscape and its elements on people^9–11^. Landscape perception and preference are dominated by limitations in human vision to a great extent^12^; however, some scholars have proposed that merits and inferiorities of the landscape could be judged by most observors^13^. Since visual landscape study emerged as a field of research in the West in the twentieth century, many scholars have examined characteristics of people’s preference for natural landscapes through a variety of subjective and objective methods^14–16^, concluding that characteristics of the landscape itself have a huge impact on visual preference^15,17,18^.

Whatever research method is adopted, most scholars use pictures as a medium for landscape research^19–21^. However, since pictures cannot fully reflect the actual viewing experience and cannot show the inherent diversity of the landscape, their value for research is limited^22^. A large number of studies have shown that social measurement research based on images correlates well with a parallel reaction in directly experiencing the represented landscape^23,24^. Compared with physical experiments, the use of pictures saves valuable research funding and provides better control over the experimental subjects. Therefore, pictures present a cost-effective medium for public visual preference research^25,26^. The use of eye-tracking technology to collect eye movement information to analyze people’s cognitive process and preference characteristics is therefore potentially useful within this framework^27^. Eye movement information mainly includes fixations, saccades and following up^28^. The corresponding indicators, such as the total duration of fixations, the average duration of fixations, the number of fixations and the average amplitude of saccades can be used to measure the attraction of stimulation target to subjects^29,30^. In addition, changes in pupil size have always been related to people’s interest in visual stimulation^31^.

Current research suggests that plant landscapes are images represented by vegetation, plant communities, and individual plants^32^, but not plant organs as landscapes. The structural features and systems of plants have been shown to have an important impact on how people interpret plants^30^. Plant growth status, plant coverage, seasonal changes, hierarchy, characteristics of individual plants and the choice of characteristic plants^15,33^ will affect viewers’ visual preference. Eye-tracking, for example, can reveal viewer visual preferences for different plant features^30^. Viewers have been shown to pay more attention to color^34^ and seasonal color changes^35^, indicating that color is one of the most tangible and influential aspects of visual preference. This provides a good way to adjust, directly guide and induce positive outcomes on individual mental states^34^. Some scholars have turned their attention to the shapes and sizes of trees^36^ and the stripes of the stems and leaves of bamboo in producing stimuli. General research has identified that the most popular seasonal landscape season is summer, and it has been observed that when observed in summer, features such as shapes, stems, leaves, flowers and fruits are very effective in producing positive stimuli^1^; however, no in-depth study has been undertaken. In effect, there is no systematic integration of people’s preferences when organs are used as plant landscapes.

The selection and application of various plants are very important in rural greening^37^, with plant selection crucial in producing positive effects, especially for rural areas. Choosing favored native plants does not just realize the function of rural greening, but also highlights local characteristics, maintain a unique regional flavor, supporting the cultural landscape and tourist attractions, thus improving the effect and quality of rural greening^38^. Consequently, the goal of this study is to select favourable native plants from the perspective of visual preference to build supporting evidence applicable to rural greening and landscape construction in China.

## Methods

### Stimuli

**Figure 1:**
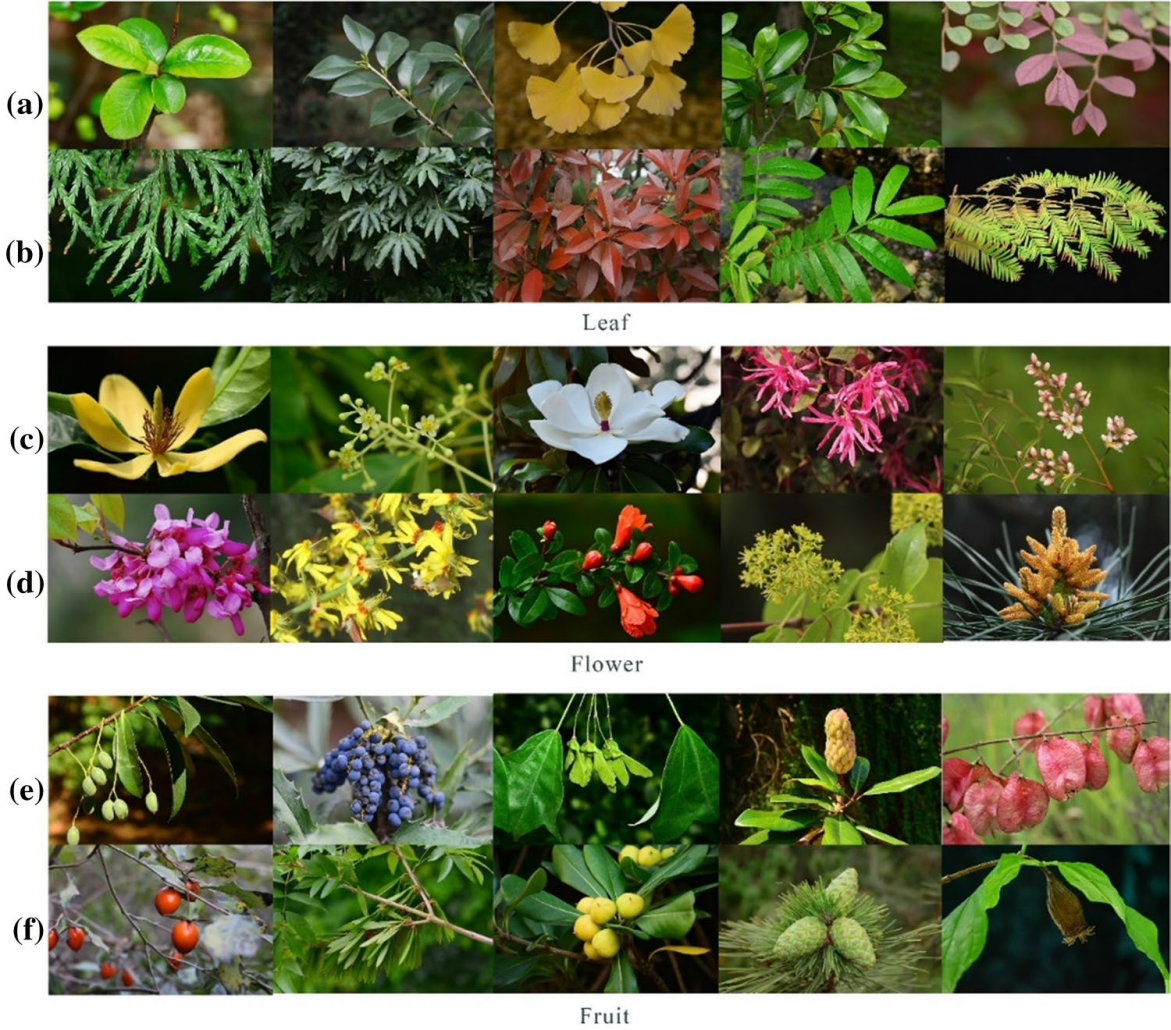
Examples of plant pictures used in the eye-tracking experiment: (**a**, **b**): leaf; (**c**, **d**): flower; (**e**, **f**): fruit. All pictures were taken by the project researcher or downloaded from the Institute of Botany, Chinese Academy of Sciences (http://www.ibcas.ac.cn) and the website of China Botanical Garden Alliance (https://www.cubg.cn).

Experimental pictures were sourced either independently or from the official website of the Institute of Botany, Chinese Academy of Sciences (http://www.ibcas.ac.cn) and the website of China Botanical Garden Alliance (https://www.cubg.cn). For the screening of plant species, a list of common native plants suitable for greening in subtropical areas was identified in the relevant literature, determined on the basis of natural distribution, then 65 native plants for greening were selected according to the opinions of botany experts. Finally, 52 native plants for greening, comprising both trees and shrubs, were obtained after filtering vines and herbs (see Table 1). For experimental photography screening, 40 kinds of plants were selected from the list, including 25 trees and 15 shrubs, according to the list of selected plants, with leaves, flowers, and fruits as classification criteria. Then pictures with leaves, flowers, or fruits of the plants as the core elements were taken and downloaded, and the background of the pictures was blurred by the depth of field mode or Photoshop CS6 software to highlight the experimental theme. To minimize tonal interference between different pictures, the picture hue was processed uniformly without changing the color of the plant itself. A total of 120 experimental pictures were obtained, and the size of all pictures was 4496 × 3000 pixels (see Fig. 1). Each experimental picture reflected some specific attributes, such as leaf color, shape, texture, and cracks; flower color, shape, and inflorescence; fruit size, color, and shape (see Table 2). For the convenience of this study, fruit with a diameter greater than 5 cm was classified as a large fruit, 3–5 cm a medium fruit, 1–3 cm a small fruit, and less than 1 cm a micro fruit. In addition, to prevent the interference of primacy effect in the experiment^23^, one picture of leaves, one of flowers, and one of fruits of other native plants which were not in the experimental list were selected as the preheating pictures before the experiment. In total, the subjects were required to browse a total of 123 pictures in the formal experiment.

**Table 1:** The native greening plants and their families selected in this study.

Number	Family	Species
1	*Cupressaceae*	*Fokienia hodginsii*
		*Metasequoia glyptostroboides*
		*Cunninghamia lanceolata*
2	*Cannabaceae*	*Celtis sinensis*
3	*Aquifoliaceae*	*Ilex rotunda*
4	*Fabaceae*	*Cercis chinensis*
5	*Elaeocarpaceae*	*Elaeocarpus glabripetalus*
6	*Pittosporaceae*	*Pittosporum tobira*
7	*Juglandaceae*	*Pterocarya stenoptera*
8	*Buxaceae*	*Buxus sinica*
9	*Hamamelidaceae*	*Liquidambar formosana*
		*Distylium racemosum*
		*Loropetalum chinense*
10	*Malvaceae*	*Hibiscus mutabilis*
11	*Calycanthaceae*	*Chimonanthus praecox*
12	*Magnoliaceae*	*Yulania liliiflora*
		*Michelia chapensis*
		*Yulania biondii*
		*Michelia maudiae*
		*Magnolia grandiflora*
		*Liriodendron chinense*
		*Michelia figo*
13	*Oleaceae*	*Fraxinus hubeiensis*
		*Ligustrum lucidum*
		*Osmanthus fragrans*
		*Jasminum mesnyi*
14	*Anacardiaceae*	*Choerospondias axillaris*
15	*Lythraceae*	*Punica granatum*
		*Lagerstroemia indica*
16	*Rosaceae*	*Cerasus campanulata*
		*Malus halliana*
		*Amygdalus persica*
		*Chaenomeles speciosa*
		*Photinia* × *fraseri*
		*Photinia serratifolia*
17	*Theaceae*	*Camellia sasanqua*
		*Camellia transarisanensis*
		*Camellia japonica*
18	*Ebenaceae*	*Diospyros rhombifolia*
19	*Pinaceae*	*Pinus thunbergii*
		*Pinus massoniana*
20	*Sapindaceae*	*Koelreuteria bipinnata*
		*Acer buergerianum*
		*Sapindus saponaria*
21	*Araliaceae*	*Fatsia japonica*
22	*Berberidaceae*	*Berberis thunbergii*
		*Nandina domestica*
		*Mahonia fortunei*
23	*Myricaceae*	*Myrica rubra*
24	*Ginkgoaceae*	*Ginkgo biloba*
25	*Lauraceae*	*Cinnamomum japonicum*
		*Cinnamomum camphora*

**Table 2:** Classification of organ characteristics and attributes of native greening plants in this study.

Features	Leaf	Flower	Fruit
Color	Green, Yellow, Red	White, Pink, Red, Yellow, Green, Purple	Brown, Black, Red, Yellow Green
Size	–	–	Large, Medium, Small, Micro
Shape	Oval, Lanceolar, Sector, Strip, Ellipse, Cordiform, Aciculiform, Jacket-shape	–	Wings, Spindle, Oval, Spherical, Kidney-shaped, spoon-shaped, Ellipse
Type	–	Corymb, Cyme, Catkin, Capitulum, Umbel, Spica, Panicle, Raceme	Samara, Drupe, Pod, Nut, Berry, Aggregate fruit, Pome, Cone, Achene, Capsule
Texture	Leather-like, Paper-like	–	–
Crack	Yes, No	–	–

### Environment

When the eye tracker is used in a real-life background, there are many uncontrollable interferences in the environment for the subjects, and the ever-changing natural light will also lead to the deviation of the pupil detection of the subjects^39,40^, which makes it difficult to ensure the reliability of the data. Therefore, the experiments were carried out in the Tourism College of CSUFT’s laboratory in Hunan Province and Changkou Village Committee’s meeting room in Fujian Province. Experimental pictures were displayed using PowerPoint projection, and the subjects sat directly in view of the projection. The indoor environment was controlled to be noiseless, dimly lit^41^ and comfortable in temperature and humidity, ensuring that the subjects were not affected by the outside world and clearly observed the experimental pictures.

### Equipment

Eye movement data were recorded using the Tobii Pro Glasses 2 wearable eye movement meter, with a lightweight design and four eye-movement cameras with resolution of 1920 × 1080@25fps. The eye movement of the subject is tracked by pupil corneal reflection and binocular dark pupil acquisition technology, which has wireless real-time observation function and supports slip compensation. If the subject touches the headset or rotates in a small range, the data quality will not be affected. The built-in gyroscope and acceleration sensor, with 0.5 precision and 100 Hz stable sampling rate, ensure extremely high data validity. In the experiment, Tobii Pro Glasses Controller software was used on one Surface tablet computer for eye movement calibration, real-time observation, eye movement video export and other operations, and the experimental pictures were shown on another computer. Eye movement data were recorded and saved to the SD card of the recording device, matched with the eye movement instrument.

### Subjects

In sociological and psychological studies, it is considered that when the effective sample size of this type of experiment reaches more than 30, it is understood as a large sample experiment, and reliable sample reliability can be obtained^42,43^. At the same time, in similar eye tracking experiments, there are generally between 30 and 90 subjects^44–46^. At present, most researchers take people of the age of college students and above as subjects^47,48^, and villagers were the first visual audience of the landscape created by native greening plants.

For this study, 59 subjects—20 males and 39 females—aged between 18 and 63 years^49^ were selected, including 33 undergraduates and postgraduates (M_Age_ = 21.79, SD_Age_ = 2.176) in CSUFT of Hunan Province and 26 villagers (M_Age_ = 47.23, SD_Age_ = 10.359) in Changkou Village of Fujian Province. All the subjects had good naked or corrected vision, no color blindness or color weakness, and all passed the eye movement calibration test used in the experiment, meeting the experimental requirements. Participants were rewarded with a cash gift of 30–50 yuan for their participation. The experiment obtained the informed consent of all subjects, and all procedures were carried out in accordance with relevant guidelines and regulations. The academic committee of CSUFT approved the study. Specific consent was not obtained because the data were analyzed anonymously.

### Pre-experiment

A preliminary experiment was conducted at Tourism College of CSUFT from March 23–24, 2021, with 12 subjects. A total of 55 species of plants were selected in the pre-experiment, with a total of 135 plant photos. 3 × 3 jigsaw puzzles were used in the material processing of the experiment, with 45 in total. According to the standards of trees, shrubs, and plant organs, there were five random jigsaw puzzles showing leaves, flowers and fruits, and each jigsaw puzzle was randomly recombined by changing the position of the plant pictures—that is, the same plant picture would be observed by subjects in different positions three times. In the experiment, participants wore eye movement equipment and sat quietly in front of the slide. A total of 12 valid sets of eye movement data were obtained.

Experimental results showed that the relative position of pictures in the jigsaw puzzle had significant influence on the participants’ response, and it was difficult to accurately obtain reliable subject preferences for local greening plants. This preliminary study showed the shortcomings of the experimental scheme, and after reflection, study and modification, the jigsaw puzzle mode was finally abandoned and replaced by a single-picture pattern as the experimental stimulus. The overall framework of the formal experiment was determined on the basis of experts' opinions.

### Procedure

Before the experiment, participants were instructed to sit down before the projection and familiarize themselves with the experimental environment. The researcher wore an eye tracker and explained the experimental process and requirements to the subjects. Subjects were then instructed to carry out eye movement calibration with eye movement calibration cards. After the calibration, three preheated pictures and 120 experimental pictures were played in turn. The experimental pictures were automatically and randomly played, and each picture was displayed for 10 s. In order to reproduce the visual preference and observation habits of the subjects in real life^44^, the subjects did not know the specific purpose of the experiment^50^; this was to prevent deliberate action due to psychological suggestion. The experimental process of each subject was about 20 min. Some subjects developed eye fatigue after long periods^51^, which led to a decrease in the number of eye movements^52^ and an overall decrease in the accuracy^53^. Therefore, subjects were allowed to signal that they wished to pause at any time during observation^49^. Another eye movement calibration would be repeated once the participant continued. After the experiment, demographic characteristics of the subjects were collected, including name, age, gender and education level, and the cash gift was issued to express gratitude.

### Data processing

The purpose of this experiment was to study the visual preference of the subjects for native greening plants from the aspects of plant characteristics, families, and species. Therefore, in the process of sorting and analyzing eye movement data, we first classified and coded plants by species, families, organs, and characteristics (see Table 2), using them as independent variables and eye movement indicators as dependent variables in SPSS 25.0 software for the analysis of variance (ANOVA). In addition, to explore the basic association structure of different species, we used total duration of fixations and number of fixations as variables for hierarchical cluster analysis on species.

Specifically, the eye movement data were pre-processed and exported by Tobii Pro Lab software. First, the AOI of each experimental picture was determined and drawn—that is, the leaf, flower, or fruit area. Then, according to the research requirements, the required eye movement index data were selected and the TSV format file was exported based on AOI. The eye movement indexes selected in this study were mainly fixation time and fixation number, including total duration of fixations, average duration of fixations, number of fixations, average amplitude of saccades and average pupil size. In the experiment, 54 valid eye movement data were obtained after unusable data with chaotic eye movement trajectory or low sampling rate were deleted. In the process of sorting out eye movement data, plant characteristics were first classified and coded, then data integration, classification, calculation, and analysis were carried out on this basis.

## Results

### Differences of eye movement indexes among different families, species, and organs

According to the results of ANOVA of eye movement data, different families, species, and organ types show significant differences in three indicators—total duration of fixations, number of fixations and average duration of fixations—and some show differences in average pupil size and average amplitude of saccades (see Table 3).

**Table 3:** Eye movement indexes ANOVA results at family, species, and organ level.

Levels	Total duration of fixations		Number of fixations		Average duration of fixations		Average pupil size		Average amplitude of saccades	
	F	*p*	F	*p*	F	*p*	F	*p*	F	*p*
Organ	281.320	0.000*	478.963	0.000*	9.308	0.000*	1.449	0.235	2.454	0.086
Species	15.129	0.000*	16.173	0.000*	1.906	0.000*	1.586	0.005*	3.591	0.000*
Family	12.824	0.000*	12.968	0.000*	2.238	0.000*	1.416	0.085	4.485	0.000*

*Indicating that there is a difference in the index of this feature.

#### Plant organs

At the level of organ types (see Table 4), leaves have the most total duration of fixations and number of fixations (MD_Leaf flower_ = 1.031, MD_Leaf fruit_ = 1.767), followed by flowers (MD_Flower fruit_ = 0.736). Fruits have the lowest fixation duration and number of fixations, but the average duration of fixations is the longest, which is significantly different from leaves (MD_Fruit leaf_ = 0.032) but has no significant difference from flowers.

**Table 4:** Descriptive analysis of eye movement indexes of different organ types.

Types	Total duration of fixations		Number of fixations		Average duration of fixations		Average pupil size		Average amplitude of saccades	
	Mean	SD	Mean	SD	Mean	SD	Mean	SD	Mean	SD
Leaf	5.492	2.376	18.43	8.144	0.322	0.236	4.146	0.879	4.95	2.154
Flower	4.462	2.503	13.69	7.810	0.352	0.265	4.101	0.866	4.94	2.224
Fruit	3.726	2.482	11.13	7.584	0.354	0.301	4.127	0.854	4.82	2.086

Precision (SD) = standard deviation of data samples.

#### Species

**Figure 2:**
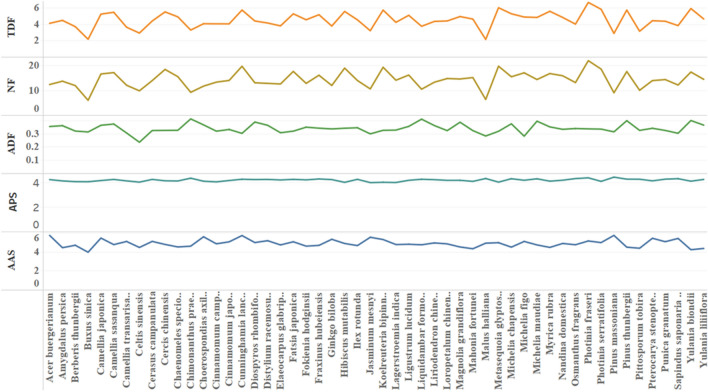
Statistical analysis of eye movement index means description of different species. Where TDF: Total duration of fixations; NF: Number of fixations; ADF: Average duration of fixations; APS: Average pupil size; AAS: Average amplitude of saccades.

Among 52 kinds of native greening plants, the average trend of total duration of fixations and number of fixations is basically the same (see Fig. 2). *Photinia* × *fraseri* (M_Duration_ = 6.692, M_Number_ = 22.02) has the largest mean value in these two indexes, *Metasequoia glyptostroboides, Photinia serratifolia, Cunninghamia lanceolata, Koelreuteria bipinnata, Hibiscus mutabilis* and *Cercis chinensis* are at the forefront, and the three indexes including average duration of fixations of *Pinus thunbergii* and *Yulania biondii* are in the top ten. *Malus halliana, Buxus sinica, Pinus massoniana, Celtis sinensis* and *Jasminum mesnyi* have less fixation duration and number of fixations. There are also significant differences in average pupil size and average amplitude of saccades among different species. *Pinus massoniana* (M_Pupil_ = 4.347, M_Saccade_ = 5.758) has the largest mean value in both indexes. In addition, the average pupil size values of *Chimonanthus praecox* and *Photinia* × *fraseri* are also very high, while those of *J. mesnyi*, *Cinnamomum camphora* and *K. bipinnata* are lower. The average amplitude of saccades of *Acer buergerianum* and *C. lanceolata* is large, and that of *B. sinica*, *Y. biondii* and *Mahonia fortunei* is small.

#### Family

**Figure 3:**
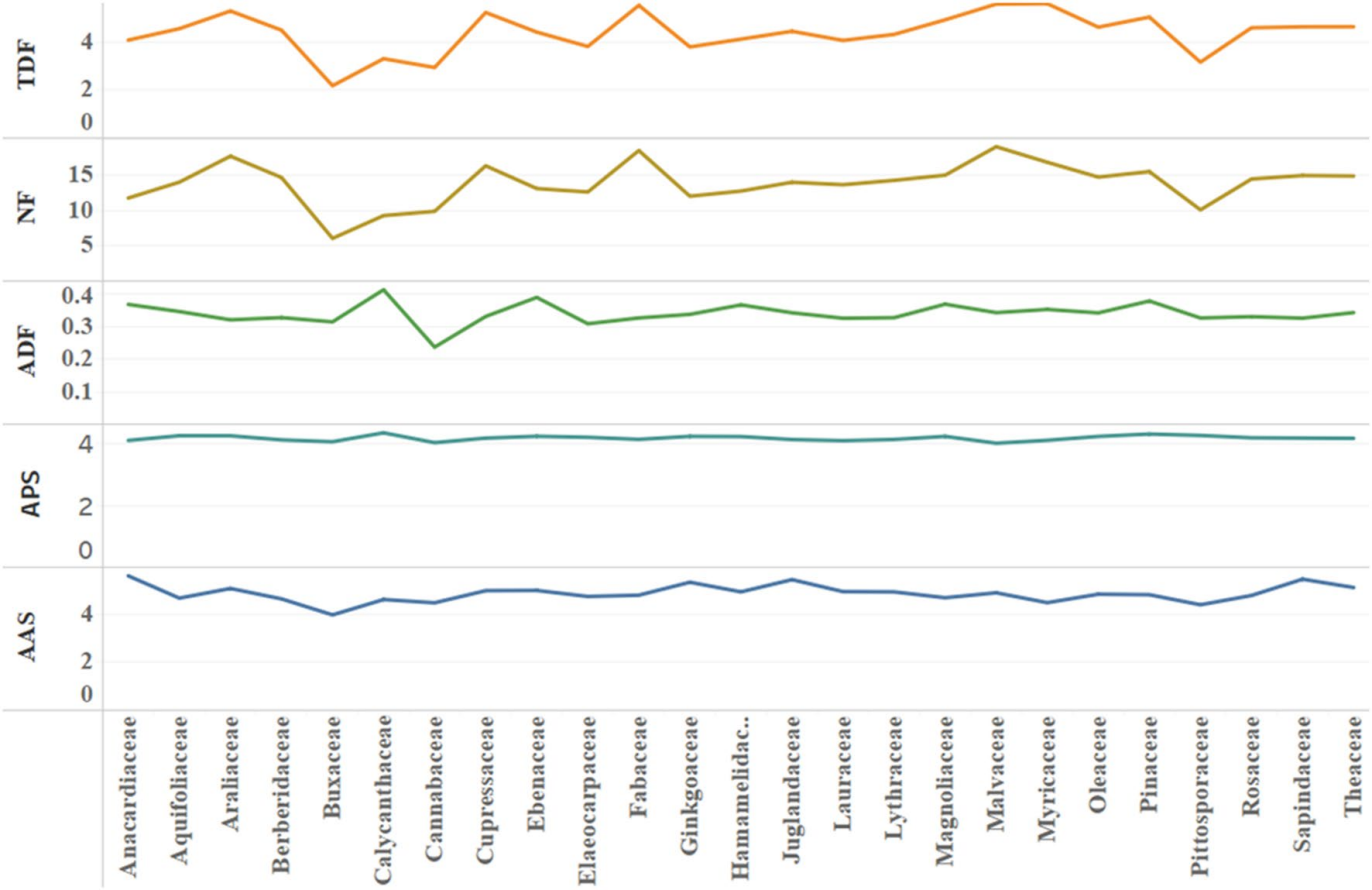
Statistical analysis of eye movement index mean description in different families. Note: TDF: Total duration of fixations; NF: Number of fixations; ADF: Average duration of fixations; APS: Average pupil size; AAS: Average amplitude of saccades.

Among the corresponding 25 families, *Malvaceae, Fabaceae, Araliaceae, Myricaceae* and *Cupressaceae* rank the top five in terms of total duration of fixations and number of fixations, while *Calycanthaceae, Ebenaceae, Pinaceae, Magnoliaceae* and *Anacardiaceae* have the highest average duration of fixations, *Buxaceae* and *Cannabaceae* are in a low state among the three indexes. *Araliaceae* has more total duration and number of fixations, while the average duration of fixations is relatively low. There are also significant differences in the average amplitude of saccades among different families. The average amplitude of saccades of *Anacardiaceae, Sapindaceae* and *Juglandaceae* is large, while that of *Buxaceae, Pittosporaceae* and *Cannabaceae* is small (see Fig. 3).

### Differences of eye movement indexes in different organs

#### Leaf

**Figure 4:**
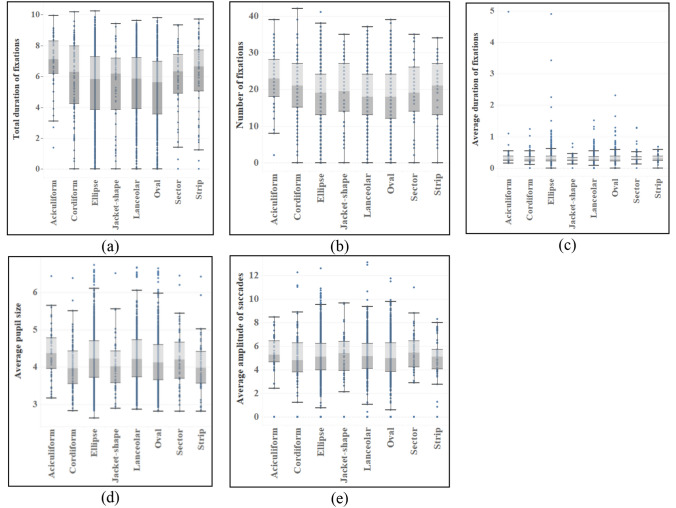
Eye movement index of different leaf shapes.

**Figure 5:**
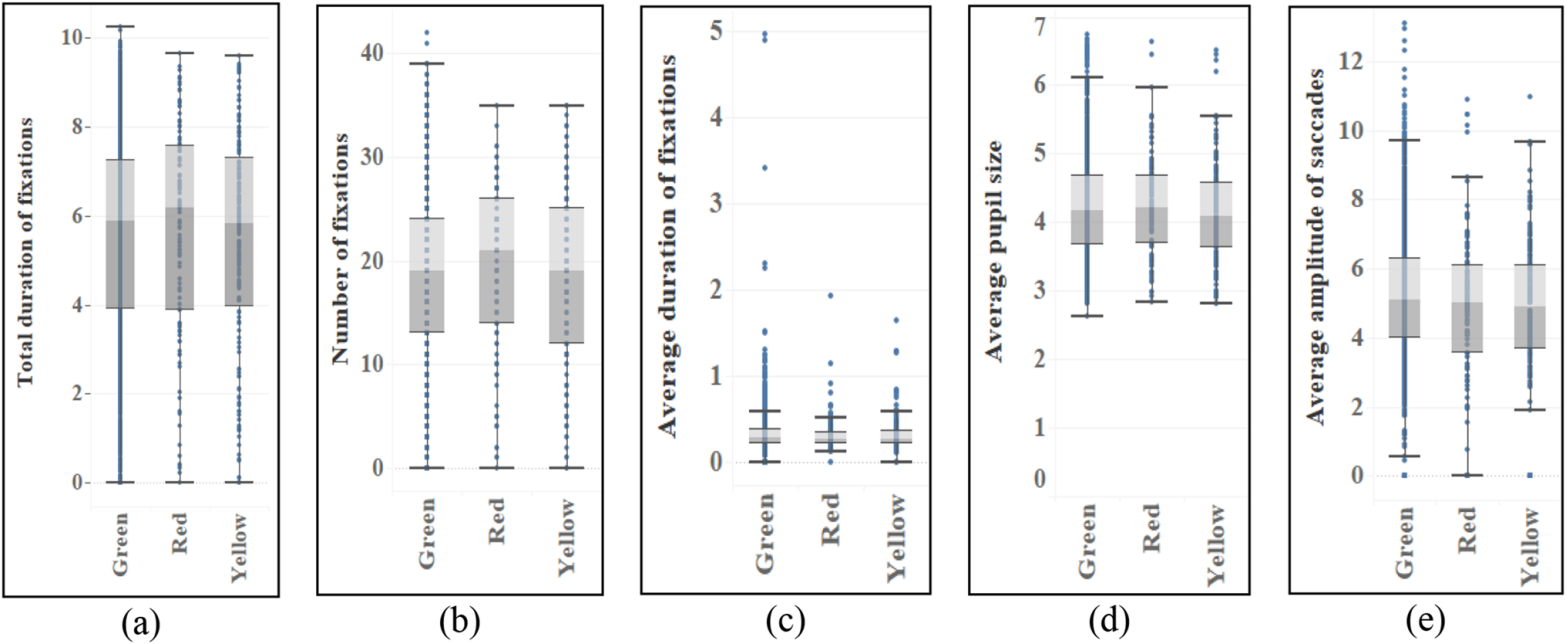
Eye movement index of different leaf colors.

**Figure 6:**
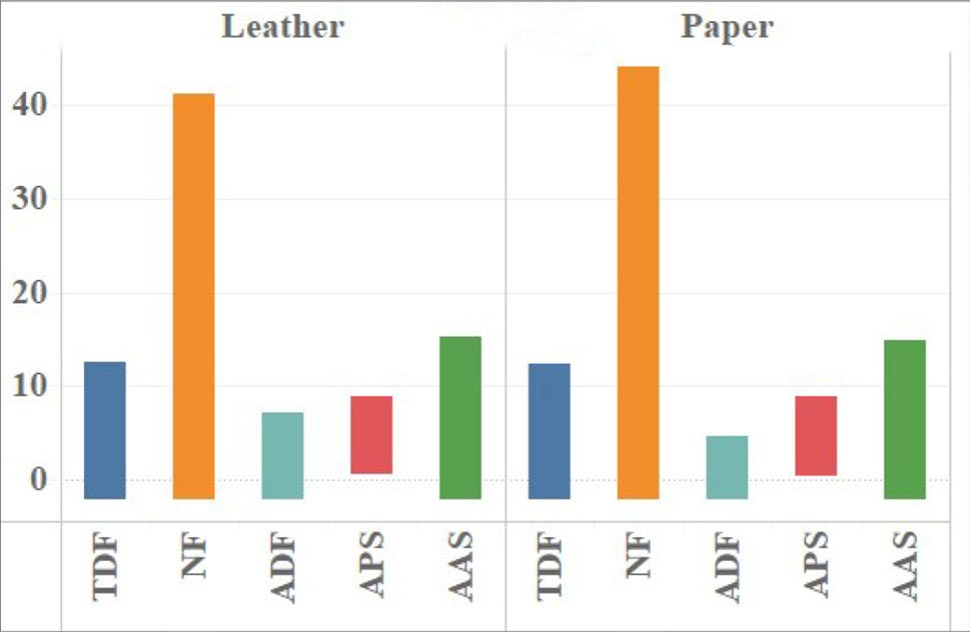
Eye movement index of different leaf texture.

**Figure 7:**
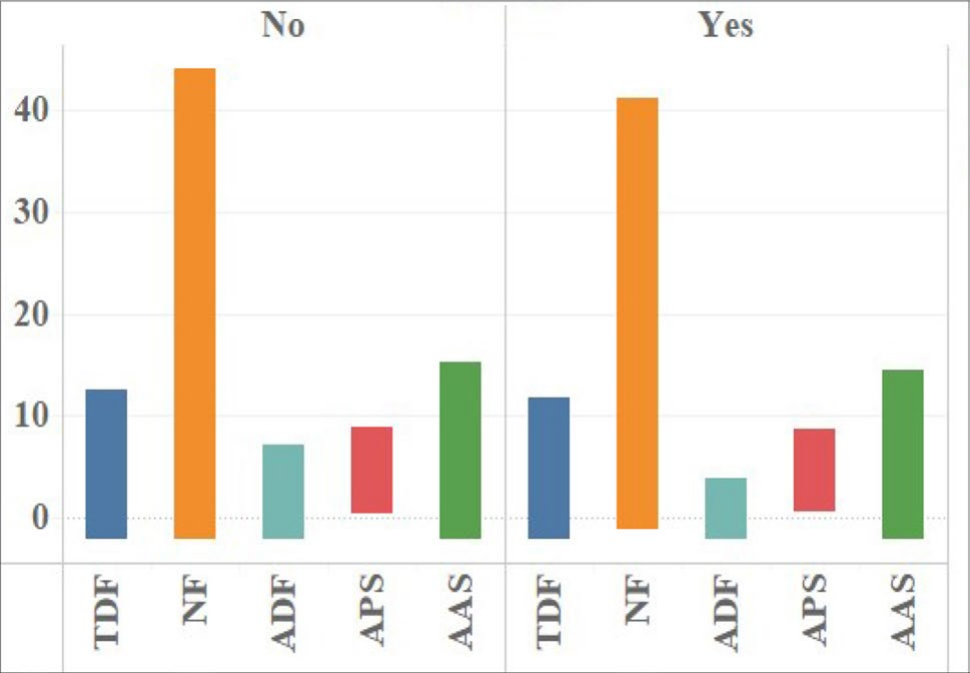
Eye movement index of leaves with and without leaf crack.

The ANOVA of eye movement data in response to different organ characteristics shows that the difference reflected by leaves is not obvious compared with those of flowers and fruits (see Table 5). There are significant differences in leaf shape in total duration and number of fixations (*p* = 0.000 < 0.05) (see Fig. 4). According to subsequent multiple comparisons, the differences between leaf shapes are from aciculiform and oval shape, among which aciculiform has the most total duration of fixations (M = 6.902) and number of fixations (M = 22.80), while oval shape (M_Duration_ = 5.263, M_Number_ = 17.85) has the least. In addition, the texture of leaves is different in the total duration of fixations (*p* = 0.020 < 0.05, MD_Texture_ = 0.254) (see Fig. 6), and there is no difference in eye movement indexes of other characteristics (see Figs. 5, 6 and 7).

**Table 5:** ANOVA results of eye movement indexes of different types of leaves.

Types	Total duration of fixations		Number of fixations		Average duration of fixations		Average pupil size		Average amplitude of saccades	
	F	*p*	F	*p*	F	*p*	F	*p*	F	*p*
Shape	4.721	0.000*	4.094	0.000*	1.849	0.074	2.006	0.051	0.767	0.615
Color	0.339	0.712	0.445	0.641	0.100	0.905	0.617	0.540	1.669	0.186
Texture	5.410	0.020*	0.947	0.331	3.381	0.066	5.561	0.018	0.946	0.331
Crack	0.677	0.411	0.463	0.496	1.562	0.212	0.913	0.339	0.176	0.675

*Indicating that there is a difference in the index of this feature.

#### Flowers

**Figure 8:**
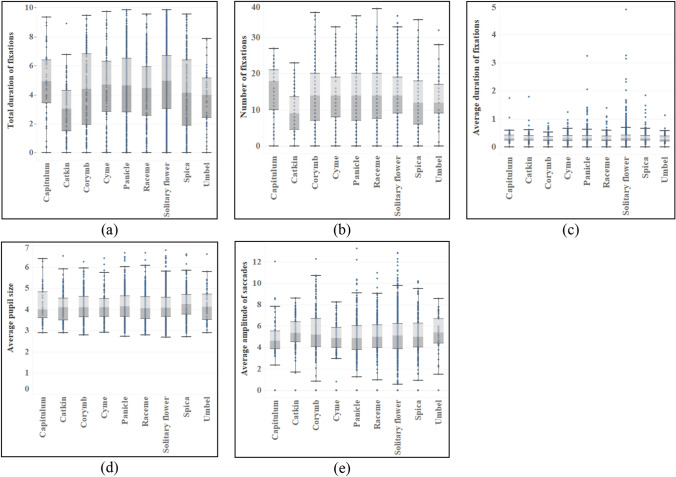
Eye movement indexes of different flower inflorescences.

**Figure 9:**
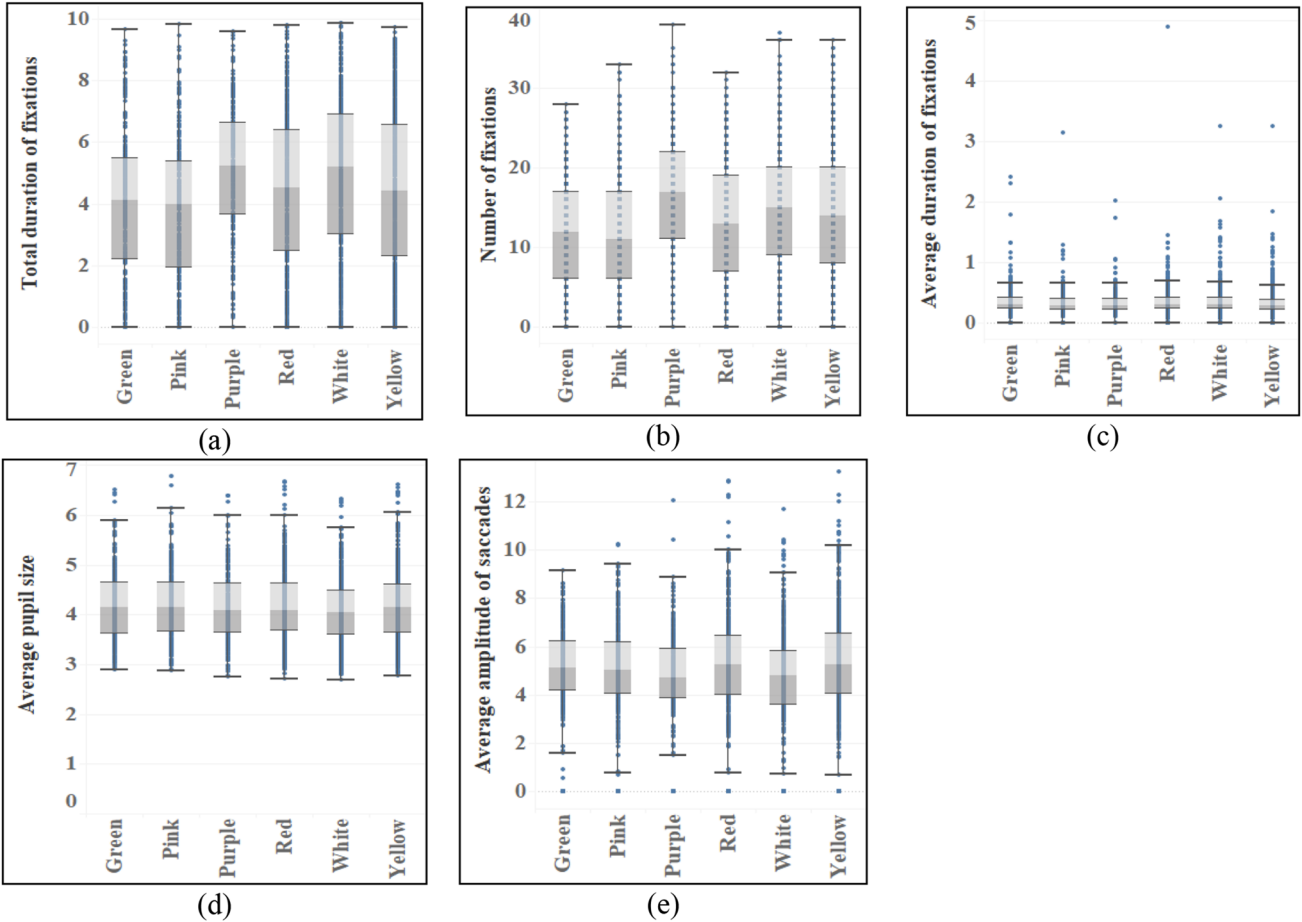
Eye movement indexes of different flower colors.

The inflorescences and colors of flowers show significant differences in the total duration of fixations and number of fixations (see Table 6), among which the capitulum (M_Duration_ = 4.900, M_Number_ = 15.46) has the largest mean value, and there are significant differences between the catkin and other inflorescences, but the mean value of the above two indexes is the smallest (M_Duration_ = 2.978, M_Number_ = 9.28). In addition, there are differences between solitary flowers, umbels and some inflorescences, and their average values are closely behind the capitulum and before the catkin (see Fig. 8). And there are differences in total duration of fixations, number of fixations and average duration of fixations between solitary flower and infinite inflorescence, but there is no difference in definite inflorescence. On the whole, solitary flower (M_Duration_ = 4.756, M_Number_ = 14.19, M_Average duration_ = 0.373) has the largest mean in three indexes, followed by definite inflorescence and infinite inflorescence. With regard to flower colors, the total duration of fixations and the order and number of fixations are basically the same and the difference is big, but there is no difference in average duration of fixations. Purple (M_Duration_ = 5.093, M_Number_ = 16.80) has the highest average value, followed by white, yellow and red. In addition, there are significant differences in the average amplitude of saccades among different colors. Multiple comparisons show that the difference mainly comes from white with the smallest average amplitude of saccades (M = 4.569). Yellow has the largest average amplitude of saccades (M = 5.119), followed by red and green (see Fig. 9).

**Table 6:** ANOVA results of eye movement indexes of different types of flowers.

Types	Total duration of fixations		Number of fixations		Average duration of fixations		Average pupil size		Average amplitude of saccades	
	F	*p*	F	*p*	F	*p*	F	*p*	F	*p*
Inflorescence	7.775	0.000*	6.388	0.000*	2.126	0.030*	0.314	0.961	1.568	0.129
Color	10.214	0.000*	13.474	0.000*	1.231	0.292	1.592	0.159	3.898	0.002*
Type	10.785	0.000*	3.343	0.036*	4.732	0.009*	0.020	0.981	0.706	0.494

*Indicating that there is a difference in the index of this feature.

#### Fruit

**Figure 10:**
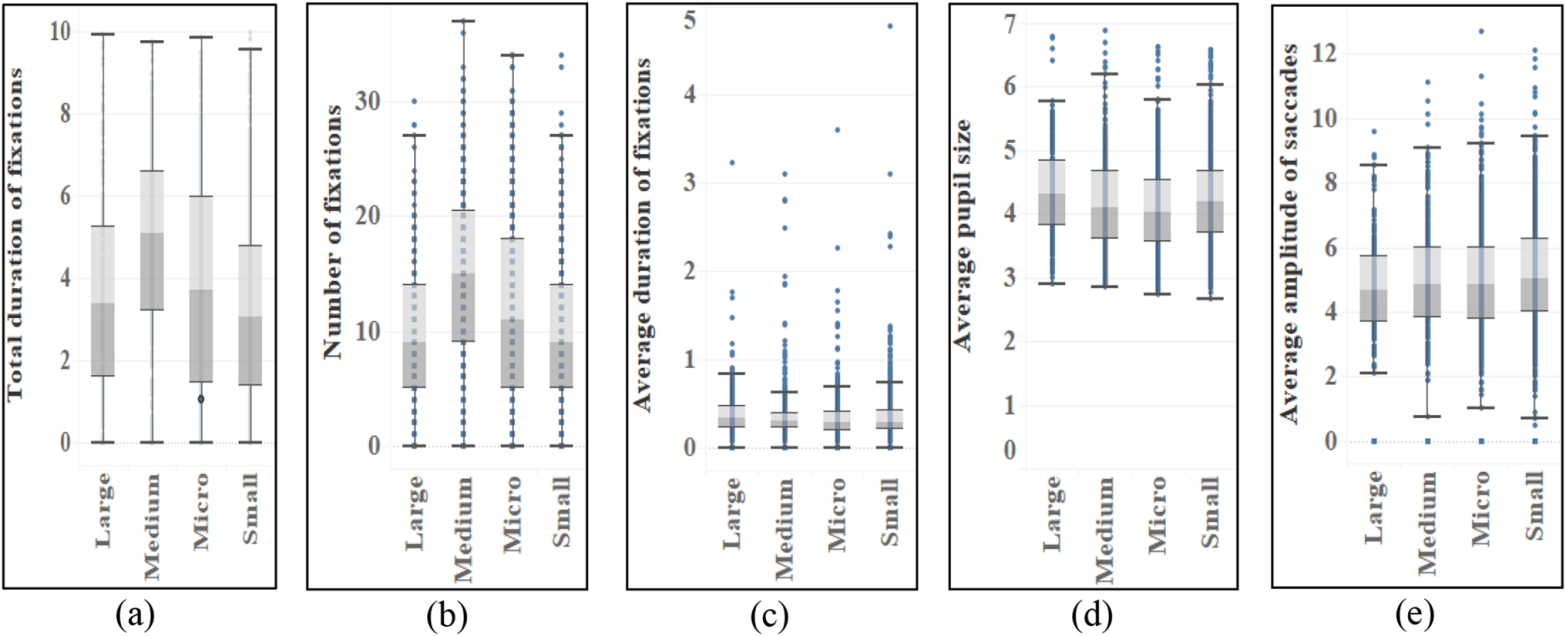
Eye movement indexes of different fruit sizes.

**Figure 11:**
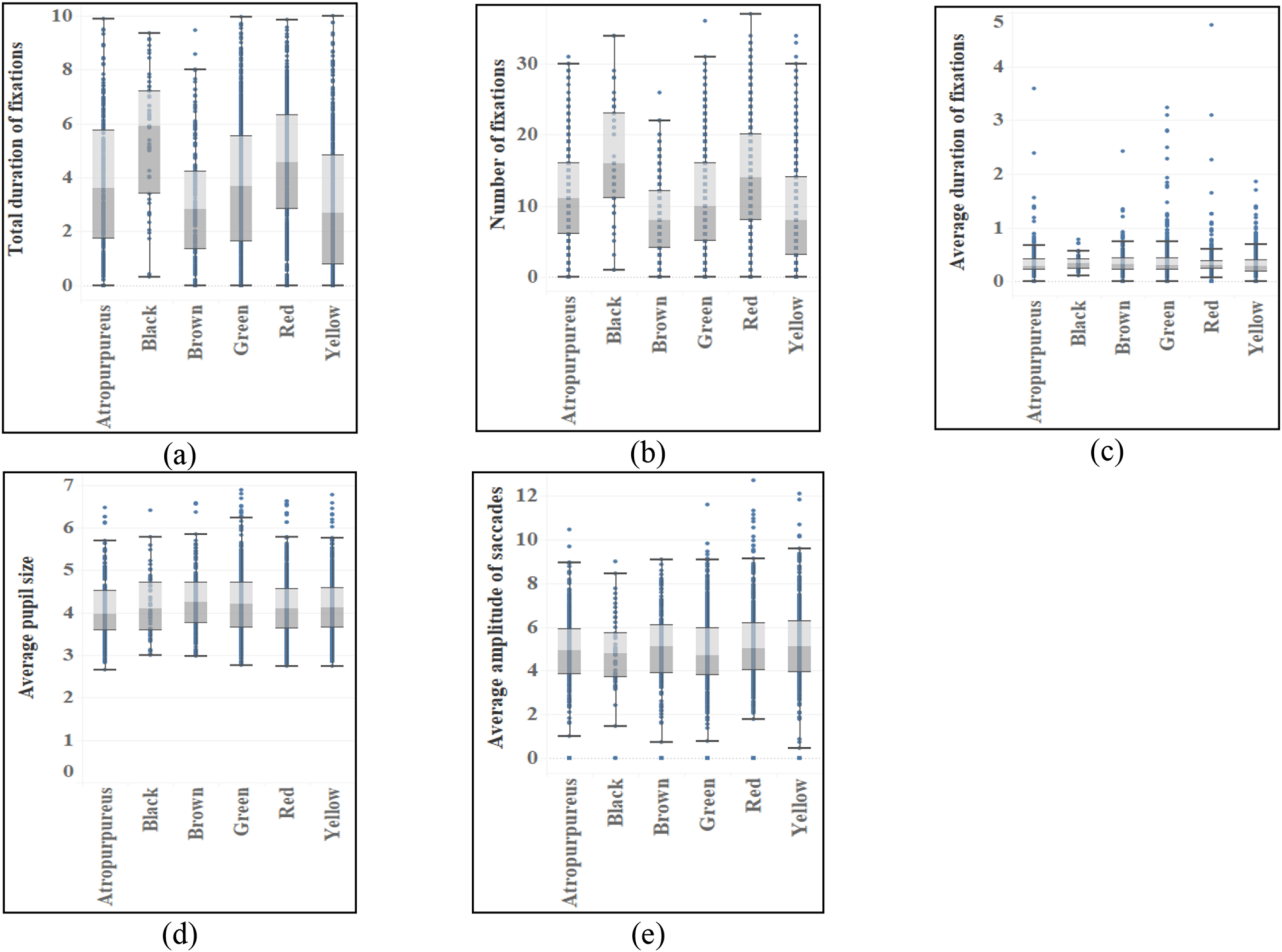
Eye movement indexes of different fruit colors.

**Figure 12:**
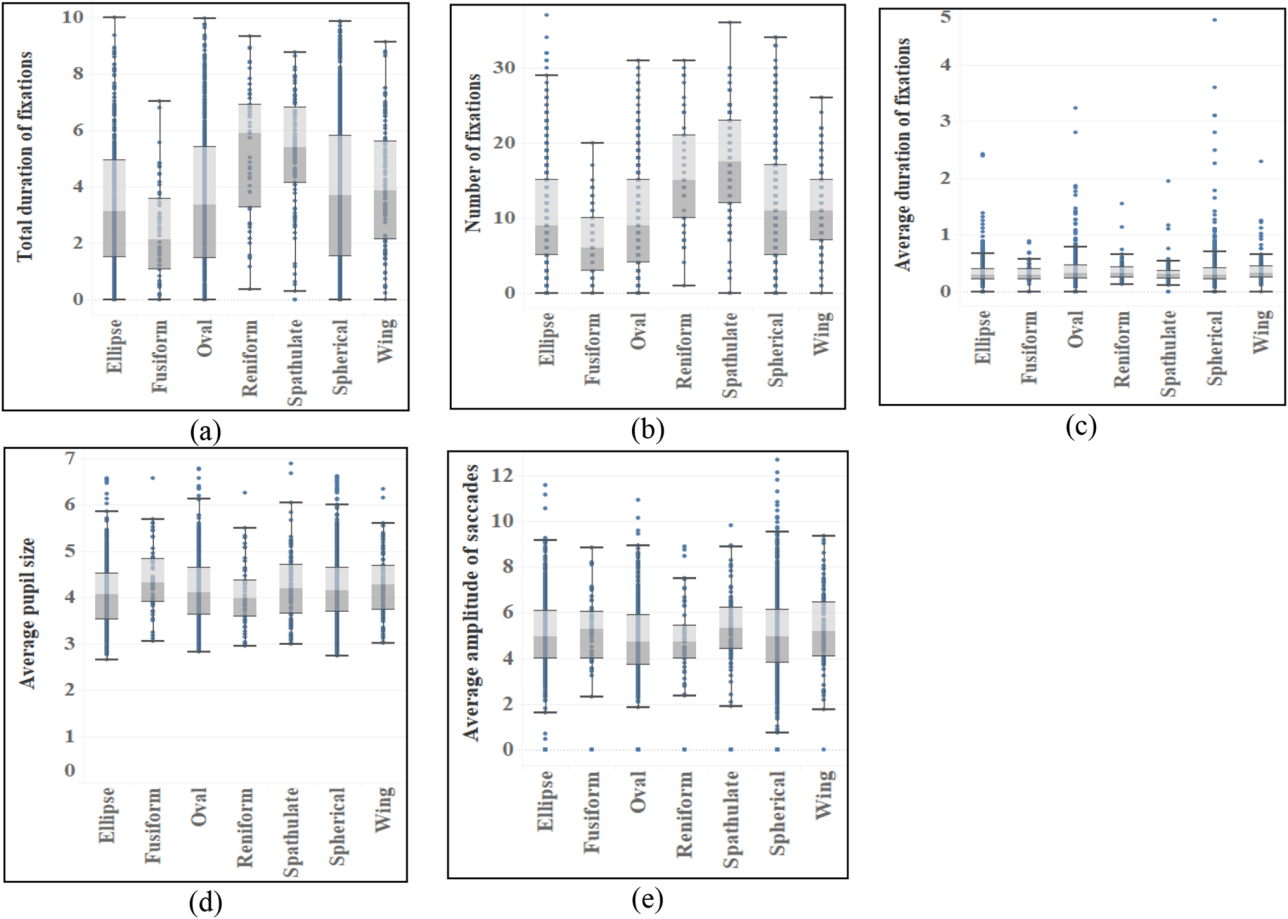
Eye movement indexes of different fruit shapes.

Virtually all other indexes show significant variance among the different characteristics of fruit (see Table 7). The mean value of two indexes of medium fruit (M_Duration_ = 4.869, M_Number_ = 14.96) was the largest, followed by micro fruit and large fruit, and the smallest was small fruit. The average pupil size of large fruit is the largest (M = 4.32), while the saccade amplitude of small fruit is the largest (M = 5.007). There are also significant differences in the average fixation time of fruits of different sizes. The average duration of fixations for large fruit (M = 0.395) is the longest (see Fig. 10). In fruit color, there are distinct differences among different colors, and the order of total fixation duration and fixation times is consistent. The mean value of black fruit (M_Duration_ = 5.419, M_Number_ = 16.93) is much higher than that of other colors, and the mean pupil size among different colors is also different. Brown (M = 4.202) has the highest mean value, followed by green and black (see Fig. 11). There are obvious differences in total fixation duration and number of fixations among fruits with different shapes, and the mean values of kidney-shaped fruits and spoon-shaped fruits are higher. At the same time, the average amplitude of saccades is also different, and the wing shape (M = 5.285) has the largest average amplitude of saccades, followed by spindle shape and spoon-shaped shape (see Fig. 12). From the aspect of fruit types, there are differences among different types of fruits, among which pome (M_Duration_ = 5.761, M_Number_ = 18.00) has the most total duration of fixations and number of fixations, with pod, samara, and berry at the forefront. There are also obvious differences in average pupil size and average amplitude of saccades among different types. The average value of achene and aggregate fruit in the former is larger, and the average value of pome (M = 5.627) in the latter is the largest.

**Table 7:** ANOVA results of eye movement indexes of different types of fruits.

Types	Total duration of fixations		Number of fixations		Average duration of fixations		Average pupil size		Average amplitude of saccades	
	F	*p*	F	*p*	F	*p*	F	*p*	F	*p*
Size	40.757	0.000*	53.740	0.000*	4.103	0.006*	8.678	0.000*	4.653	0.003*
Color	28.194	0.000*	38.794	0.000*	2.125	0.060	2.635	0.022*	2.208	0.051
Shape	14.752	0.000*	20.651	0.000*	1.211	0.298	1.548	0.159	2.433	0.024*
Type	14.226	0.000*	18.283	0.000*	1.824	0.059	2.400	0.011*	4.065	0.000*

*Indicating that there is a difference in the index of this feature.

### Differences of eye movement indicators in different educational backgrounds

The primary focus from the results is on eye movement differences between participants with university level education and above, and people with high school education and below. The data showed that there were significant differences in five eye movement indexes (*p* = 0.000 < 0.05), and the total duration of fixations, number of fixations, average pupil size and average amplitude of saccades of people with bachelor's education and above were much higher than those with high school education and below (MD_Duration_ = 1.237, MD_Number_ = 5.57, MD_Pupil_ = 0.341, MD_Saccade_ = 1.175), but the average duration of fixations is the opposite (see Table 8).

**Table 8:** ANOVA results of eye movement indexes of different educational background.

Varieties	Total duration of fixations		Number of fixations		Average duration of fixations		Average pupil size		Average amplitude of saccades	
	Mean	SD	Mean	SD	Mean	SD	Mean	SD	Mean	SD
University level and above	5.109	2.517	16.89	8.267	0.321	0.181	4.264	0.725	5.425	2.119
High school education and below	3.872	2.442	11.32	7.519	0.371	0.348	3.950	0.990	4.250	2.020

Precision (SD) = standard deviation of data samples.

### Cluster analysis

**Figure 13:**
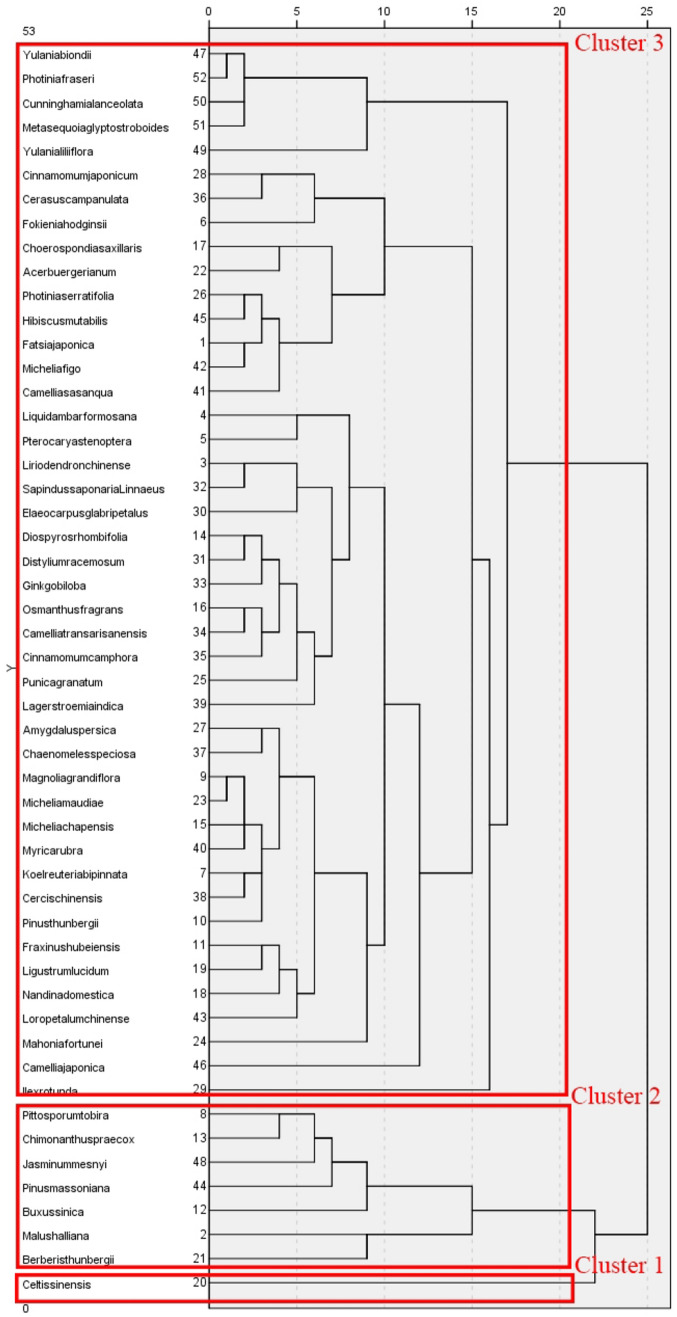
Clustering analysis of total duration of fixations.

**Figure 14:**
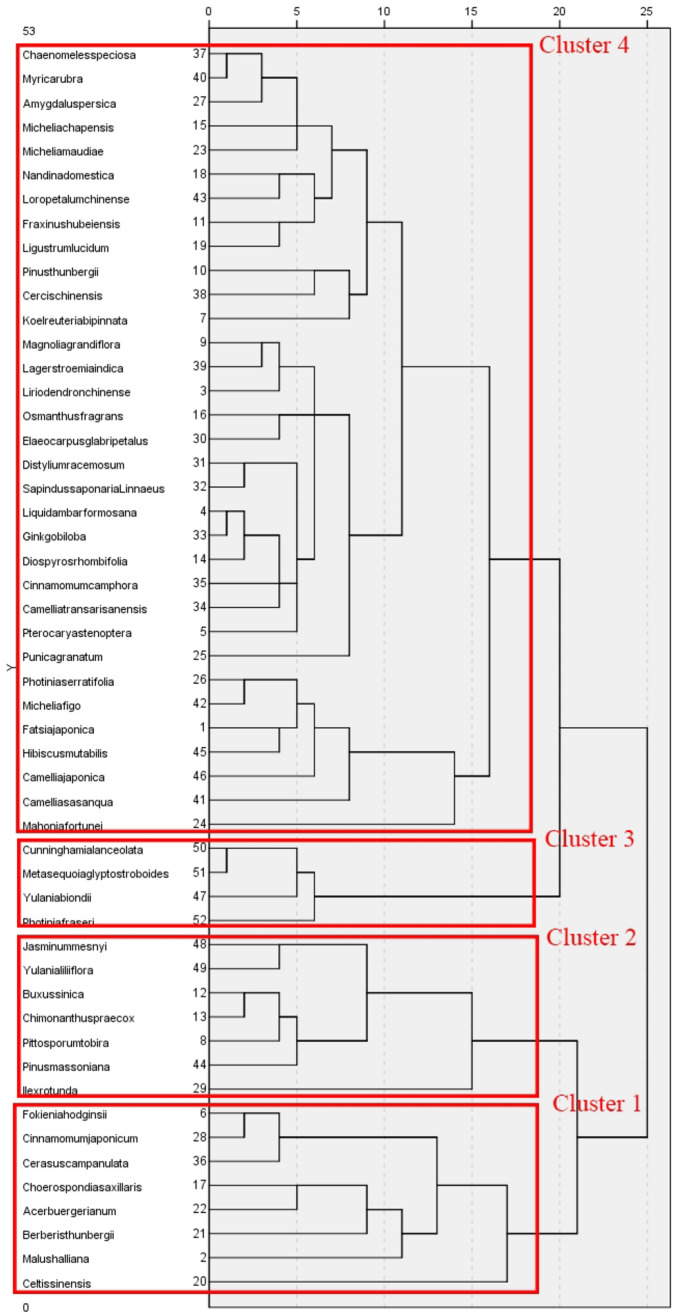
Clustering analysis of number of fixations.

Cluster analysis of the total duration of fixations and number of fixations of the species involved in the study showed that all species could be divided into three clusters in terms of total duration of fixations. The first cluster contains only *C. sinensis*; the second cluster includes *Pittosporum tobira, C. praecox, J. mesnyi, P. massoniana, B. sinica*, *M. halliana* and *Berberis thunbergia*; and the remaining species are all in the third cluster (see Fig. 13). In terms of number of fixations, all species can be divided into four clusters. The first cluster contains *Fokienia hodginsii, Cinnamomum japonicum, Cerasus campanulata, Choerospondias axillaris, A. buergerianum, B. thunbergii, M. halliana* and *C. sinensis*; the second cluster contains *J. mesnyi, Yulania liliiflora, B. sinica, C. praecox, P. tobira, P. massoniana* and *Ilex rotunda*; the third cluster includes *C. lanceolata, M. glyptostroboides, Y. biondii* and *Photinia* × *fraseri*, and the rest of the species are included in the fourth cluster (see Fig. 14).

## Discussion

Five eye movement indexes, total duration of fixations, average duration of fixations, number of fixations, average amplitude of saccades and average pupil size have been selected for analysis and summary. At present, academics in China and abroad have and applied and conducted explorations in many different fields using these indexes, concluding that total duration of fixations and number of fixations can reflect the visual preference of subjects to a certain extent. People prefer attractive stimuli with longer fixation time and more fixations^54^. Average pupil size is also related to preference^31^ as a measure, reflecting people’s sensitivity to stimulus materials^30^. On the contrary, the longer the average duration of fixations, the larger the information conveyed by stimulus, and the more time subjects need to spend acquiring and processing information^55,56^. The average amplitude of saccades reflects the range of information acquired. The larger the amplitude, the more vivid the picture featured, and the easier it is for subjects to directly reach the target area^19^.

The results show that there are significant differences in visual preferences among different plant organ types. People prefer leaves visually, followed by flowers, with fruits having the lowest visual preference. This is different from the research results in the majority of literature examined^34,57^, as there is variance between established facts and research preferences^58^. It was found in a study that although test subjects expressed their preference for plants with larger or colorful flowers, only one-third of the plants were blooming in the selected material^59^. Compared with flowers and fruits, processing leaves information^60^ requires less time and effort; by contrast, flowers and fruits convey a huge amount of visual information, which people take longer to process. In terms of species, *Photinia* × *fraseri, M. glyptostroboides, P. serratifolia, C. lanceolata, K. bipinnata, H. mutabilis, C. chinensis, P. thunbergii* and *Y. biondii* have higher overall preference, and the characteristics of *P. massoniana* and *C. lanceolata* are distinct and easily catch people’s eyes. Furthermore, at the botany family level, *Malvaceae, Fabaceae, Araliaceae, Myricaceae* and *Cupressaceae* have stronger visual appeal, and *Anacardiaceae, Juglandaceae* and *Sapindaceae* are more prominent.

The most prominent feature of plants is the differentiation of flowers and fruits in different seasons^1^. Therefore, although leaves have higher preference weight compared with flowers and fruits, the overall difference between leaves with different characteristics is indistinct. Obvious differences in eye movement indexes exist between different leaf shapes and textures, which influence preference. The influence of leaf shape is consistent with the research results of some academics^59^. Aciculiform, strip, cordiform, sector and jacket-shaped leaves are more attractive to people, and leather-like leaves have higher visual preference than paper-like leaves. However, little to no difference exists in preferences between different colors and whether leaves are cracked or not cracked. Vringer et al.^61^ also concluded that the color of flowers is more popular than the color of leaves.

People’s preference for flowers with different inflorescences and colors is very significant. In terms of inflorescence types, they prefer solitary flowers, finite inflorescences, and infinite inflorescences. Specifically, capitulum, cyme, panicle, corymb, and raceme are more attractive to people than the other three inflorescences. As for color, purple is the most preferred color, followed by white, yellow, and red, among which yellow and red are distinct and attention drawing, while white is the opposite. This is consistent with the conclusion of Rahnema et al.^34^, whose results show that red ranks first, followed by purple, orange and colorful (a combination of any of the other colors). The results of a study conducted in the United States also support this conclusion^62^.

Compared with leaves and flowers, all the characteristics of fruit deviate from each other, and different characteristics correlate strongly with fruit viewing results. First, there is a visual preference for medium-sized fruits, followed by micro-sized and large-sized fruits. Viewers need greater attention to acquire and process information conveyed by fruits of different sizes, and large-sized fruits can stimulate vision more effectively. Regarding color, black fruits are visually favored, followed by red, atropurpureus, green, yellow, and brown, with brown producing a stronger visual stimulation. In the analysis of other colors, white, green and yellow—which have high contrast—can create a lively atmosphere^63^; red provides an optimistic mood; and purple, like brown, may also stimulate vision^64^. In fruit shapes, kidney-shaped and spoon-shaped produced greater stimuli than other shapes, followed by wing and spherical shapes, among which the characteristics of wing and spoon-shaped are prominent and easily attract attention. From the aspect of fruit types, pome, pod, samara, and berry are paid more attention to, among which pome has the most prominent characteristics, but achene and aggregate fruit have greater visual stimulation.

There are significant differences in the duration and number of fixations among people with different educational levels. In the research process, most scholars hold the same view and think that apart from educational level^16,65,66^, group differences such as age^67^, professional background^49^ and cultural background^68,69^ will cause differences in visual preference characteristics and browsing patterns. Except for the average duration of fixations, the other four indicators show that participants with bachelor’s education and above rank much higher than those with high school education and below, which may indicate that people with a lower education level need more time to acquire and process the visual information conveyed, while higher education levels react in greater levels of sensitivity to leaves, flowers and fruits of plants, and can identify and lock the areas of interest effectively.

## Conclusion

Through eye-tracking technology, viewer visual preference was examined through a sample of students and local villagers for native greening plants commonly used in subtropical areas in this study. Results found that the attraction of leaves was higher than that of flowers and fruits, and aciculiform, strip and leather-like leaves were preferred. Among the characteristics of flowers, capitulum and purple were more attractive. For fruits, medium fruits with a diameter of 3–5 cm were preferred, while black, red, and green fruits, and kidney-shaped and pome fruits received more attention. On the whole, the plants of *Malvaceae, Fabaceae, Araliaceae, Myricaceae and Cupressaceae* have higher visual preference, and *Photinia* × *fraseri, M. glyptostroboides, C. lanceolata, P. serratifolia, K. bipinnata, H. mutabilis, C. chinensis, P. thunbergii* and *Y. biondii* have stronger visual attraction, being the first choice for greening with native plants. This study is expected to provide scientific reference for the selection of native plants, improve rural greening technology, provide new scientific research ideas, and promote the improvement of settled rural environments. It will also contribute to the development of green and livable villages and towns in China.

Some limitations exist in this study that must be discussed. As only leaves, flowers and fruits are used as stimulating material from various plant organs, the research depth is limited. In future research, we should consider expanding our scope by taking individuals, populations, and communities as experimental subjects for further research, while adopting more evidence-based methodology in material selection. At the same time, the sample size of this study is limited, and follow-up studies must consider expanding the sample size to increase statistical reliability in the future. In addition, the study selected two varying groups of students and local villagers as participants, without discussing in depth the differences between them and the consequent effects on statistical results. Effective comparison methods should be adopted in follow-up research, and larger cases and sample sizes should be selected to explore the scope of the materials, to obtain a more universal conclusion.

## Supplementary Information

Supplementary Information.
