# Supplementary material for: Study on selection of native greening plants based on eye-tracking technology
Source: Sci Rep. 2022 Jan 20;12:1092. doi: 10.1038/s41598-022-05114-0 (PMC8776756; doi:10.1038/s41598-022-05114-0)
Supplement: Supplementary file 1 — Supplementary Information. [file 41598_2022_5114_MOESM1_ESM.docx]

**Appendix**

| **Species** | **Total duration of fixations** | **Number of fixations** | **Average duration of fixations** | **Average pupil size** | **Average amplitude of saccades** |
| --- | --- | --- | --- | --- | --- |
| *Photinia fraseri* | 6.692 | 22.019 | 0.338 | 4.293 | 5.166 |
| *Metasequoia glyptostroboides* | 6.046 | 19.796 | 0.320 | 4.027 | 4.988 |
| *Yulania biondii* | 5.946 | 17.444 | 0.401 | 4.103 | 4.248 |
| *Photinia serratifolia* | 5.856 | 18.630 | 0.336 | 4.049 | 5.000 |
| *Pinus thunbergii* | 5.761 | 17.648 | 0.400 | 4.160 | 4.514 |
| *Cunninghamia lanceolata* | 5.761 | 19.778 | 0.304 | 4.179 | 5.738 |
| *Koelreuteria bipinnata* | 5.756 | 19.377 | 0.327 | 3.962 | 5.320 |
| *Myrica rubra* | 5.606 | 16.832 | 0.353 | 4.027 | 4.493 |
| *Hibiscus mutabilis* | 5.588 | 19.037 | 0.343 | 4.009 | 4.905 |
| *Cercis chinensis* | 5.536 | 18.494 | 0.327 | 4.056 | 4.805 |
| *Camellia sasanqua* | 5.489 | 17.213 | 0.375 | 4.164 | 4.787 |
| *Michelia chapensis* | 5.305 | 15.531 | 0.376 | 4.274 | 4.519 |
| *Fatsia japonica* | 5.300 | 17.694 | 0.321 | 4.250 | 5.085 |
| *Camellia japonica* | 5.262 | 16.661 | 0.365 | 4.116 | 5.476 |
| *Fraxinus hubeiensis* | 5.189 | 16.154 | 0.343 | 4.148 | 4.704 |
| *Ligustrum lucidum* | 5.117 | 16.231 | 0.357 | 4.121 | 4.825 |
| *Magnolia grandiflora* | 4.964 | 14.648 | 0.388 | 4.071 | 4.537 |
| *Chaenomeles speciosa* | 4.924 | 15.570 | 0.327 | 4.119 | 4.539 |
| *Michelia figo* | 4.907 | 17.130 | 0.283 | 4.060 | 5.120 |
| *Nandina domestica* | 4.859 | 15.944 | 0.335 | 4.078 | 4.906 |
| *Michelia maudiae* | 4.855 | 14.421 | 0.396 | 4.268 | 4.753 |
| *Yulania liliiflora* | 4.669 | 14.500 | 0.367 | 4.243 | 4.383 |
| *Mahonia fortunei* | 4.659 | 15.217 | 0.325 | 4.010 | 4.350 |
| *Fokienia hodginsii* | 4.571 | 12.880 | 0.351 | 4.091 | 4.625 |
| *Ilex rotunda* | 4.563 | 14.018 | 0.347 | 4.097 | 4.683 |
| *Amygdalus persica* | 4.495 | 13.759 | 0.362 | 4.073 | 4.461 |
| *Pterocarya stenoptera* | 4.454 | 14.006 | 0.343 | 4.077 | 5.454 |
| *Loropetalum chinense* | 4.427 | 14.826 | 0.325 | 4.118 | 4.862 |
| *Cerasus campanulata* | 4.421 | 14.000 | 0.326 | 4.127 | 5.123 |
| *Diospyros rhombifolia* | 4.419 | 13.112 | 0.390 | 4.154 | 5.003 |
| *Punica granatum* | 4.391 | 14.401 | 0.326 | 4.238 | 5.091 |
| *Liriodendron chinense* | 4.371 | 13.358 | 0.363 | 4.171 | 4.963 |
| *Lagerstroemia indica* | 4.256 | 14.143 | 0.329 | 3.996 | 4.793 |
| *Distylium racemosum* | 4.175 | 12.883 | 0.366 | 4.165 | 5.198 |
| *Acer buergerianum* | 4.136 | 12.417 | 0.356 | 4.188 | 5.755 |
| *Choerospondias axillaris* | 4.087 | 11.769 | 0.368 | 4.023 | 5.612 |
| *Cinnamomum camphora* | 4.075 | 13.377 | 0.320 | 3.947 | 4.870 |
| *Cinnamomum japonicum* | 4.069 | 14.065 | 0.333 | 4.080 | 5.080 |
| *Osmanthus fragrans* | 4.037 | 13.186 | 0.341 | 4.286 | 4.773 |
| *Sapindus saponaria Linnaeus* | 3.857 | 12.217 | 0.305 | 4.251 | 5.438 |
| *Elaeocarpus glabripetalus* | 3.821 | 12.621 | 0.309 | 4.123 | 4.752 |
| *Ginkgo biloba* | 3.800 | 12.025 | 0.338 | 4.205 | 5.346 |
| *Liquidambar formosana* | 3.773 | 10.528 | 0.412 | 4.231 | 4.768 |
| *Berberis thunbergii* | 3.731 | 11.981 | 0.322 | 4.064 | 4.719 |
| *Camellia transarisanensis* | 3.657 | 12.124 | 0.307 | 4.075 | 5.117 |
| *Chimonanthus praecox* | 3.309 | 9.278 | 0.414 | 4.346 | 4.624 |
| *Jasminum mesnyi* | 3.230 | 10.648 | 0.300 | 3.914 | 5.558 |
| *Pittosporum tobira* | 3.167 | 10.093 | 0.326 | 4.181 | 4.404 |
| *Celtis sinensis* | 2.944 | 9.883 | 0.237 | 3.987 | 4.488 |
| *Pinus massoniana* | 2.901 | 9.093 | 0.316 | 4.347 | 5.758 |
| *Buxus sinica* | 2.183 | 6.056 | 0.315 | 4.057 | 3.983 |
| *Malus halliana* | 2.161 | 6.389 | 0.284 | 4.278 | 4.938 |

**Table 1**. Mean results of eye movement indexes of different species.

| **Family** | **Total duration of fixations** | **Number of fixations** | **Average duration of fixations** | **Average pupil size** | **Average amplitude of saccades** |
| --- | --- | --- | --- | --- | --- |
| *Myricaceae* | 5.606 | 16.832 | 0.353 | 4.027 | 4.493 |
| *Malvaceae* | 5.588 | 19.037 | 0.343 | 4.009 | 4.905 |
| *Fabaceae* | 5.536 | 18.494 | 0.327 | 4.056 | 4.805 |
| *Araliaceae* | 5.300 | 17.694 | 0.321 | 4.250 | 5.085 |
| *Cupressaceae* | 5.237 | 16.333 | 0.331 | 4.097 | 4.994 |
| *Pinaceae* | 5.046 | 15.509 | 0.379 | 4.207 | 4.825 |
| *Magnoliaceae* | 4.932 | 15.007 | 0.369 | 4.176 | 4.699 |
| *Theaceae* | 4.643 | 14.886 | 0.343 | 4.112 | 5.126 |
| *Sapindaceae* | 4.641 | 14.958 | 0.326 | 4.127 | 5.473 |
| *Oleaceae* | 4.625 | 14.734 | 0.342 | 4.158 | 4.847 |
| *Rosaceae* | 4.600 | 14.458 | 0.331 | 4.137 | 4.793 |
| *Aquifoliaceae* | 4.563 | 14.018 | 0.347 | 4.097 | 4.683 |
| *Berberidaceae* | 4.502 | 14.680 | 0.328 | 4.049 | 4.651 |
| *Juglandaceae* | 4.454 | 14.006 | 0.343 | 4.077 | 5.454 |
| *Ebenaceae* | 4.419 | 13.112 | 0.390 | 4.154 | 5.003 |
| *Lythraceae* | 4.324 | 14.272 | 0.328 | 4.118 | 4.942 |
| *Hamamelidaceae* | 4.126 | 12.755 | 0.367 | 4.171 | 4.944 |
| *Anacardiaceae* | 4.087 | 11.769 | 0.368 | 4.023 | 5.612 |
| *Lauraceae* | 4.073 | 13.652 | 0.325 | 4.000 | 4.954 |
| *Elaeocarpaceae* | 3.821 | 12.621 | 0.309 | 4.123 | 4.752 |
| *Ginkgoaceae* | 3.800 | 12.025 | 0.338 | 4.205 | 5.346 |
| *Calycanthaceae* | 3.309 | 9.278 | 0.414 | 4.346 | 4.624 |
| *Pittosporaceae* | 3.167 | 10.093 | 0.326 | 4.181 | 4.404 |
| *Cannabaceae* | 2.944 | 9.883 | 0.237 | 3.987 | 4.488 |
| *Buxaceae* | 2.183 | 6.056 | 0.315 | 4.057 | 3.983 |

**Table 2**. Mean results of eye movement indexes of different families.

| **Characteristic** | | **Total duration of fixations** | **Number of fixations** | **Average duration of fixations** | **Average pupil size** | **Average amplitude of saccades** |
| --- | --- | --- | --- | --- | --- | --- |
| Leaf Shape | Aciculiform | 6.902 | 22.80 | 0.409 | 4.373 | 5.268 |
|  | Strip | 6.046 | 19.80 | 0.320 | 4.027 | 4.988 |
|  | Heart-shape | 5.924 | 20.28 | 0.311 | 3.935 | 4.743 |
|  | Sector | 5.914 | 19.28 | 0.351 | 4.159 | 5.324 |
|  | Jacket-shape | 5.529 | 19.57 | 0.287 | 4.003 | 5.063 |
|  | Ellipse | 5.453 | 18.10 | 0.332 | 4.184 | 4.922 |
|  | Lanceolar | 5.446 | 18.36 | 0.310 | 4.152 | 5.037 |
|  | Oval | 5.263 | 17.85 | 0.314 | 4.128 | 4.885 |
| Leaf Color | Red | 5.669 | 19.08 | 0.327 | 4.193 | 4.864 |
|  | Green | 5.486 | 18.42 | 0.322 | 4.149 | 4.983 |
|  | Yellow | 5.444 | 18.15 | 0.329 | 4.080 | 4.669 |
| Leaf Texture | Leather | 5.575 | 18.55 | 0.329 | 4.177 | 4.922 |
|  | Paper | 5.321 | 18.18 | 0.309 | 4.082 | 5.018 |
| Leaf Crack | No | 5.506 | 18.39 | 0.325 | 4.152 | 4.960 |
|  | Yes | 5.365 | 18.79 | 0.303 | 4.091 | 4.895 |
| Inflorescence | Capitulum | 4.900 | 15.46 | 0.346 | 4.131 | 4.825 |
|  | Solitary flower | 4.756 | 14.19 | 0.374 | 4.098 | 4.920 |
|  | Cyme | 4.557 | 14.04 | 0.338 | 4.093 | 4.715 |
|  | Panicle | 4.538 | 13.68 | 0.360 | 4.082 | 4.744 |
|  | Corymb | 4.356 | 14.17 | 0.306 | 4.122 | 5.264 |
|  | Raceme | 4.272 | 14.46 | 0.315 | 4.061 | 4.884 |
|  | Spica | 4.191 | 12.41 | 0.352 | 4.174 | 5.035 |
|  | Umbel | 3.720 | 12.83 | 0.313 | 4.132 | 5.205 |
|  | Catkin | 2.978 | 9.28 | 0.334 | 4.076 | 5.344 |
| Flower Type | Solitary flower | 4.756 | 14.19 | 0.374 | 4.098 | 4.920 |
|  | Definite inflorescence | 4.557 | 14.04 | 0.338 | 4.093 | 4.715 |
|  | Indefinite inflorescence | 4.239 | 13.30 | 0.338 | 4.104 | 4.973 |
| Flower Color | Purple | 5.093 | 16.80 | 0.338 | 4.150 | 4.759 |
|  | White | 4.847 | 14.44 | 0.370 | 4.010 | 4.569 |
|  | Yellow | 4.472 | 13.95 | 0.337 | 4.096 | 5.119 |
|  | Red | 4.419 | 13.16 | 0.359 | 4.103 | 5.096 |
|  | Green | 4.046 | 11.86 | 0.367 | 4.144 | 5.022 |
|  | Pink | 3.804 | 12.18 | 0.339 | 4.172 | 4.977 |
| Fruit Size | Medium | 4.869 | 14.96 | 0.379 | 4.076 | 4.679 |
|  | Micro | 3.798 | 11.72 | 0.326 | 4.022 | 4.730 |
|  | Large | 3.581 | 9.66 | 0.395 | 4.327 | 4.549 |
|  | Small | 3.240 | 9.49 | 0.354 | 4.175 | 5.007 |
| Fruit Shape | Reniform | 5.263 | 15.94 | 0.378 | 4.083 | 4.678 |
|  | Spathulate | 5.145 | 16.90 | 0.330 | 4.097 | 5.030 |
|  | Wing | 4.028 | 11.42 | 0.397 | 4.288 | 5.285 |
|  | Spherical | 3.771 | 11.36 | 0.347 | 4.141 | 4.816 |
|  | Oval | 3.580 | 9.91 | 0.375 | 4.103 | 4.582 |
|  | Ellipse | 3.341 | 10.43 | 0.342 | 4.075 | 4.925 |
|  | Fusiform | 2.389 | 6.94 | 0.327 | 4.325 | 5.006 |
| Fruit Type | Pome | 5.761 | 18.00 | 0.354 | 4.109 | 5.627 |
|  | Pod | 4.894 | 16.06 | 0.330 | 4.139 | 5.315 |
|  | Samara | 4.600 | 14.04 | 0.369 | 4.209 | 4.944 |
|  | Berry | 4.481 | 13.82 | 0.375 | 4.064 | 4.853 |
|  | Nut | 4.251 | 12.50 | 0.385 | 4.215 | 5.427 |
|  | Cone | 3.952 | 10.63 | 0.389 | 4.045 | 4.059 |
|  | Aggregate | 3.581 | 9.66 | 0.395 | 4.327 | 4.549 |
|  | Drupe | 3.348 | 10.12 | 0.329 | 4.098 | 4.888 |
|  | Capsule | 3.335 | 10.03 | 0.349 | 4.083 | 4.737 |
|  | Achene | 3.309 | 9.28 | 0.414 | 4.346 | 4.624 |
| Fruit Color | Black | 5.419 | 16.93 | 0.348 | 4.167 | 4.655 |
|  | Red | 4.507 | 14.10 | 0.353 | 4.067 | 5.073 |
|  | Atropurpureus | 3.801 | 11.50 | 0.366 | 4.001 | 4.747 |
|  | Green | 3.745 | 10.86 | 0.374 | 4.184 | 4.674 |
|  | Yellow | 3.008 | 8.92 | 0.318 | 4.144 | 4.832 |
|  | Brown | 2.986 | 8.28 | 0.364 | 4.202 | 4.799 |

**Table 3**. Mean results of eye movement index of different plant organ characteristics.
